# The effects of needle and warm needle on hemodynamic responses at different acupoints in patients with knee osteoarthritis

**DOI:** 10.3389/fbioe.2025.1690336

**Published:** 2025-10-21

**Authors:** Ahui Ni, Qianqian Chen, Jiayi Huang, Caiyu Liu, Jiayi Zhu, Yating Yu, Xiaoling Wang, Yih-Kuen Jan

**Affiliations:** ^1^ College of Rehabilitation Medicine, Fujian University of Traditional Chinese Medicine, Fuzhou, China; ^2^ College of Rehabilitation Medicine, Nanjing Medical University, Nanjing, China; ^3^ Rehabilitation Engineering Lab, Department of Kinesiology and Community Health, University of Illinois at Urbana-Champaign, Champaign, IL, United States

**Keywords:** knee osteoarthritis, acupuncture, acupoint, near-infrared spectroscopy, hemodynamic responses

## Abstract

**Background:**

Acupuncture, as a non-pharmacological alternative therapy, has been widely used in China for the clinical treatment of knee osteoarthritis (KOA). Different acupuncture methods and acupoint selections are two critical factors influencing clinical efficacy. Although multiple studies have confirmed the efficacy of acupuncture in treating KOA, its underlying physiological mechanisms remain unclear.

**Objective:**

This study aimed to explore hemodynamic responses in KOA patients before and after interventions with different acupuncture methods and acupoint combinations, monitoring hemodynamic changes during acupuncture using near-infrared spectroscopy (NIRS).

**Methods:**

A total of 24 KOA patients received needle and warm needle interventions at SP10 (*xuehai*) and ST34 (*liangqiu*). NIRS was used to monitor real-time changes in oxygenated hemoglobin (HbO_2_), deoxy-hemoglobin (Hb), total hemoglobin (tHb), and oxygenation during acupuncture.

**Results:**

Compared with needle, warm needle significantly increased HbO_2_ (P < 0.05) and oxygenation levels in KOA patients (P < 0.01). Both methods reduced Hb concentrations compared to baseline, with a greater decrease at ST34 than at SP10 (P < 0.05). However, during treatment, the warm needle showed an increase in the Hb concentration, especially in SP10.

**Conclusion:**

Our study provides the first evidence that different combinations of acupuncture methods and acupoint selections can significantly affect hemodynamic responses. Warm needle significantly improved local blood flow and oxygen delivery capacity in KOA patients, maintaining these improvements longer than needle intervention. Furthermore, warm needle at SP10 demonstrated superior efficacy in improving oxygen metabolism compared to ST34​.

## 1 Introduction

Knee osteoarthritis (KOA) is a common chronic disease associated with knee joint degeneration that affects elderly people (Jang et al., 2021). Globally, KOA is the 11th leading cause of disability, impacting approximately 3.8% of the world’s population (Avendaño-Coy et al., 2020). The incidence of KOA has risen steadily in recent years, driven by aging populations and increasing rates of obesity (Hunter and Bierma-Zeinstra, 2019). It is a primary cause of knee dysfunction and disability in older adults, resulting in substantial economic and social burdens (Liu et al., 2018; Liu et al., 2021). Current clinical guidelines recommend non-steroidal anti-inflammatory drugs (NSAIDs), glucosamine, and chondroitin sulfate as first-line pharmacological interventions for KOA management (Gelber, 2015). While these agents demonstrate analgesic efficacy, their clinical utility is constrained by dose-dependent adverse effects, including gastrointestinal toxicity and potential hepatotoxicity, with additional concerns regarding inconclusive evidence for sustained therapeutic benefits (Fernández-Martín et al., 2021; Magni et al., 2021). In recent years, acupuncture has gained popularity as a nonpharmacological treatment for KOA, offering benefits such as effective symptom relief, minimal side effects, and low cost (Chen et al., 2021).

Numerous clinical trials and published meta-analyses have demonstrated the safety and effectiveness of acupuncture in treating KOA. Acupuncture has been shown to reduce pain symptoms and improve daily functioning in KOA patients (Liu C et al., 2024; Liu X et al., 2024). Warm needle therapy, a variation of acupuncture, has also proven effective for KOA (Jiang et al., 2022; Jin and Guan, 2022). Studies suggest that acupuncture can provide pain relief and functional improvement lasting 3–6 months in patients with KOA (Chen et al., 2024). Compared to other traditional Chinese medicine treatments, warm needle shows superior overall efficacy for KOA (Jin and Guan, 2022). Traditional needling involves inserting thin, solid needles into specific body areas (such as acupoints), while warm needling incorporates the use of moxa poles placed on top of the needles to generate heat (Hunter and Harris, 2021). A recent network meta-analysis compared seven acupuncture treatments for KOA revealed that silver needle > floating needle > needle knife > fire needle > warm needle > conventional acupuncture > electroacupuncture, with warm needle outperforming conventional acupuncture (Ma et al., 2023). Both warm needle and needle are commonly used to treat KOA; despite robust clinical evidence demonstrating the superior efficacy of warm needle therapy over needle in KOA management, the physiological mechanisms underlying its advantages, particularly its hemodynamic effects on microvascular perfusion and metabolic regulation, remain insufficiently characterized.

In addition to acupuncture methods, the precision of acupoint selection constitutes a pivotal determinant of therapeutic outcomes in KOA (Kolasinski et al., 2020; Öztürk and Yetişir, 2023; Yetişir and Öztürk, 2023). The biochemical changes in the acupoint area induced by acupuncture stimulation are also an important step in initiating the acupuncture effect. Currently, the selection of acupoints is largely based on the clinician’s experience and traditional acupuncture theories (Mi et al., 2023). However, there is no universally accepted standard for acupoint selection in treating KOA. At present, it lacks a specific biological basis for different acupoint selection. Current evidence suggests that SP10 (*xuehai*) and ST34 (*liangqiu*) are commonly used acupoints for KOA treatment (Wang et al., 2022; Yu and Keong, 2022). SP10, located along the Spleen Meridian of Foot-Taiyin, and ST34, belonging to the Stomach Meridian of Foot-Yangming, are anatomically positioned within the vastus medialis and vastus lateralis musculatures, respectively (World Health Organization, 2008). Some studies suggest that patients with KOA exhibit weakness in the vastus medialis and stiffness in the vastus lateralis (Chang et al., 2021; Li et al., 2024). However, the therapeutic specificity of acupoint selection remains unquantified by objective biomarkers, and no studies have systematically compared the hemodynamic responses, such as localized blood flow variations and oxygen transport-metabolism, induced by different acupoint stimulations in KOA management.

Near-infrared spectroscopy (NIRS) is a clinically valuable, portable, and noninvasive tool for monitoring hemodynamic parameters, including concentrations of oxyhemoglobin (HbO_2_), deoxy-hemoglobin (Hb), and total hemoglobin (tHb) (Boushel and Piantadosi, 2000; Scholkmann et al., 2014). Current NIRS investigations in KOA have predominantly focused on detecting cerebral hemodynamic alterations associated with pain processing, while its utilization in characterizing lower extremity musculoskeletal hemodynamics has been scarcely investigated (Öztürk et al., 2021). Yang et al. (2024) demonstrated that Fu’s subcutaneous needling (FNS) intervention significantly enhanced blood flow and oxygenation in the vastus lateralis muscle of affected limbs, revealing that acupuncture-induced hemodynamic changes may play an essential role in therapeutic effects. However, no studies have systematically characterized hemodynamic variations elicited by different acupuncture methods and acupoint selections in KOA patients.

The purpose of this study was to investigate the effects of needle and warm needling on localized hemodynamic responses in KOA patients when applied to two distinct acupoints: SP10 and ST34. Specifically, the research objectives are to (1) evaluate the hemodynamic responses induced by needle versus warm needle interventions through quantitative measurements of HbO_2_, Hb, tHb, and oxygenation and (2) investigate the hemodynamic responses and their dynamic changes, respectively, induced by different acupuncture methods (needle and warm needle) and acupoint selections (SP10 and ST34). To our knowledge, this is the first study to comprehensively explore the combined effects of different acupuncture methods and acupoint selections on hemodynamic responses in KOA patients.

## 2 Materials and methods

A 2 × 2 factorial crossover trial was implemented, incorporating two acupuncture methods (needle and warm needle) and two acupoints (SP10 and ST34). The study employed four acupuncture schemes: (A) needle at SP10; (B) warm needle at SP10; (C) needle at ST34; (D) warm needle at ST34. The sample size calculation was based on a pilot study involving eight patients with KOA. All participants received four different protocols (A, B, C, and D) sequentially, with an interval of 5 to 7 days between each protocol. Since previous studies have demonstrated that acupuncture can induce significant changes in local tissue HbO_2_ levels (Gao et al., 2023; Yang et al., 2024), HbO_2_ was selected as the primary outcome measure. With an effect size of 0.25, a statistical power of 0.8, an alpha level of 0.05, and accounting for four measurement time points, the required sample size was determined to be 24 using G*Power software (ANOVA, repeated measures, within–between interaction). The four distinct acupuncture protocols generated 24 unique treatment sequences via full permutation (Table 1). To control for potential order effects and ensure balance across sequences, the 24 participants were randomly assigned to the 24 sequences using a computer-generated randomization list. This ensured that each participant had an equal probability of receiving any of the possible sequences.

**TABLE 1:** Specific test orders of four acupuncture schemes.

Subject	Order of four schemes of acupuncture
#1	A, B, D, C
#2	D, C, B, A
#3	A, C, B, D
#4	D, A, C, B
#5	C, D, A, B
#6	C, A, D, B
#7	B, C, A, D
#8	A, C, D, B
#9	D, C, A, B
#10	B, D, C, A
#11	C, A, B, D
#12	B, D, A, C
#13	A, B, C, D
#14	D, B, C, A
#15	B, A, D, C
#16	C, D, B, A
#17	B, A, C, D
#18	C, B, D, A
#19	A, D, B, C
#20	D, A, B, C
#21	C, B, A, D
#22	B, C, D, A
#23	D, B, A, C
#24	A, D, C, B

The study comprised 24 patients diagnosed with KOA who met the specified inclusion criteria: (1) meeting the clinical diagnostic criteria for KOA set by the American College of Rheumatology (Altman et al., 1986); (2) aged 40–75 years; (3) grade 2 or 3 KOA based on the Kellgren and Lawrence grading scale; (4) Numeric Rating Scale (NRS) score greater than 3 during daily activities; (5) absence of skin lesions at the acupuncture sites. Exclusion criteria included the following: (1) history of joint or musculoskeletal injuries in the lower limbs aside from KOA; (2) radiographic evidence of bone bridge formation or joint stiffness; (3) presence of acute inflammation or edema-associated pain; (4) previous surgery or arthroscopic interventions; (5) severe lower limb deformities; (6) ​​interventions within 4 weeks prior to the study that might affect study outcomes, including oral or injectable NSAIDs, hormone therapy, intra-articular injections, physical therapy, or acupuncture​​.

### 2.1 Near-infrared spectroscopy

Dynamic changes in hemodynamic parameters, including HbO_2_, Hb, and tHb, were continuously monitored using the PortaLite™ near-infrared spectroscopy system (Artinis Medical Systems, Elst, Netherlands) (Klop et al., 2023; Maidan et al., 2017). The oxygenation status ([HbO_2_] − [Hb]) was calculated as the difference between HbO_2_ and Hb concentrations. We used the three probes to improve signal robustness; they examined acupoints and the tissue area around them. The system employed dual-wavelength near-infrared light (760 nm and 850 nm) with wireless Bluetooth connectivity, sampling at 10 Hz to capture real-time hemoglobin concentration fluctuations at acupoints SP10 (2 inches above the medial base of the patella) and ST34 (2 inches above the lateral base of the patella) during needle and warm needling interventions. Ambient light interference was minimized using black shielding fabric, with data acquisition and analysis performed via Oxysoft software (v3.0.52, Artinis Medical Systems).

**FIGURE 1:**
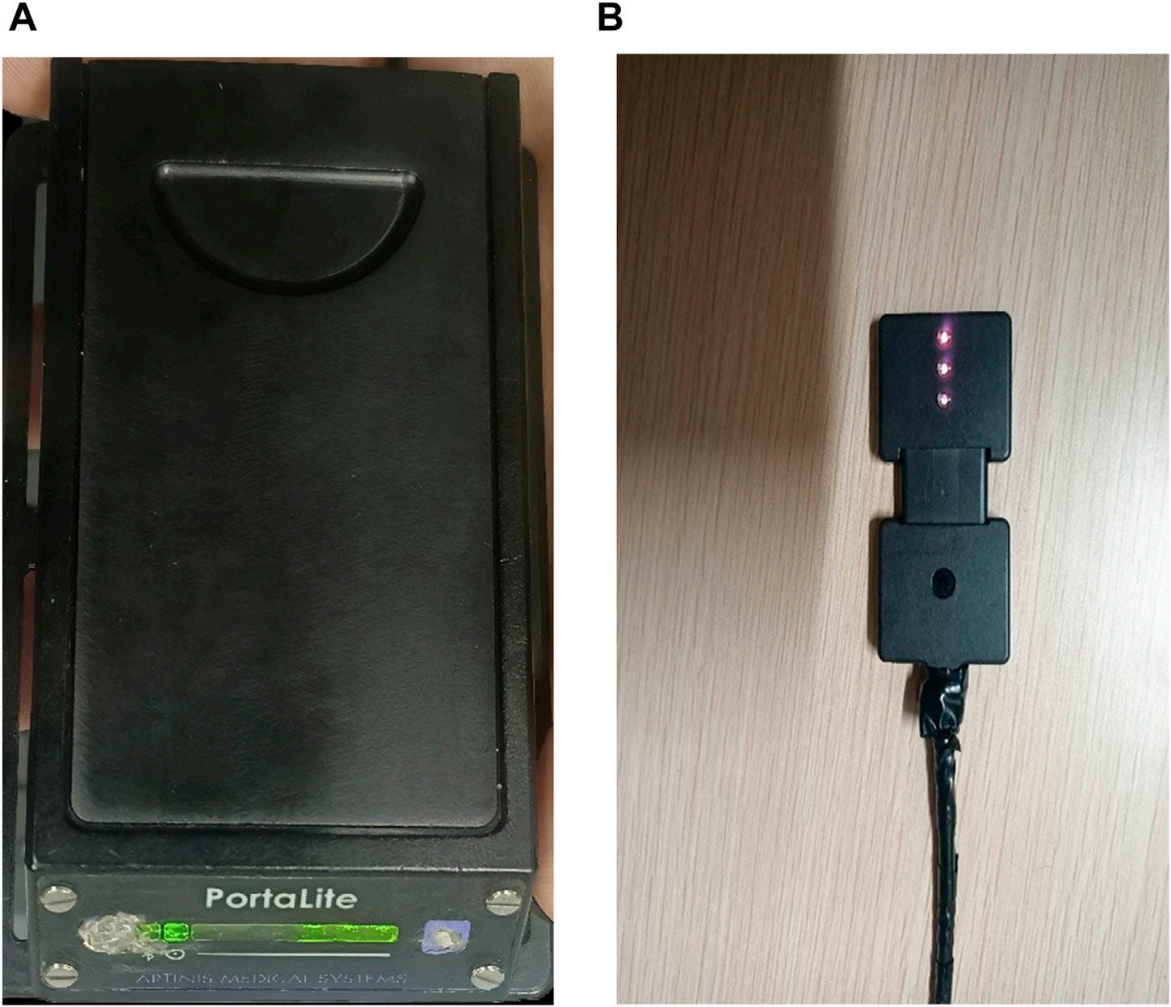
Photographs of NIRS device. **(A)** PortaLite™ system case. **(B)** PortaLite™ system incorporates three LEDs and a receiver.

The PortaLite™ system employed in this study comprised three light-emitting diode (LED) transmitters (Tx) and a single high-sensitivity PIN diode receiver (Rx) (Figures 1A,B). The Tx–Rx distances were set at 30, 35, and 40 mm. Hemoglobin concentration underwent changes. The concentrations ([HbO_2_] and [Hb]) were calculated using the modified Lambert–Beer law (MBLL) equation based on wavelength-dependent absorption differences between HbO_2_ and Hb (Scholkmann et al., 2014). A moving Gaussian filter with 0.5-s bandwidth was applied post-acquisition to attenuate high-frequency noise and physiological artifacts. The concentration changes (post-acupuncture–pre-acupuncture around acupoints, including skin and muscle hemodynamic responses) in Δ[HbO_2_], Δ[Hb], Δ[tHb], and Δoxygenation (Δ [HbO_2_]-Δ [Hb]) were reported in this study.

### 2.2 Procedure

**FIGURE 2:**
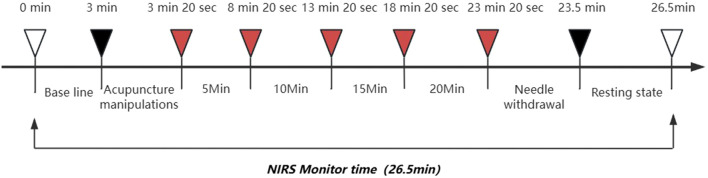
Experimental process diagram.

All participants provided written informed consent before the study. The experiments were conducted in a temperature-controlled environment (24–26 °C) at the Sports Rehabilitation Laboratory of Fujian University of Traditional Chinese Medicine. Patients were acclimated by lying supine for ≥ 5 min without speaking or unnecessary muscle movements. NIRS signals were collected for 26.5 min during the experiment, including baseline measurement for 3 min and acupuncture manipulations for 20 s (needle was 10 s of needle insertion and 10 s of needle twisting; warm needle was 10 s of needle insertion and 10 s of adding moxa stick), acupuncture treatment for 20 min (20 min was divided into four 5-min periods , each period of needle including 10 s of needle twisting and 4 min 50 s of rest, while the warm needle did not twist the needle and rest), followed by 10 s of needle out, and finally 3 min of rest (Figure 2). Marks of SP10 and ST34 acupoints were made 2 inches above the inner and outer ends of the patellar base. During the formal measurement, participants adopted a supine position with both knees flexed 15°–20°. A foam roller, diameter 9.6 cm and length 30 cm, was placed under the knees to relax the muscles and reduce muscle contraction.

The selected acupoints were thoroughly disinfected before treatment. Sterile disposable needles (Huatuo brand, Suzhou Medical Supplies Factory Co., Ltd., GB 2024-1994) sized 0.30 × 50–60 mm were inserted to a depth of 25–30 mm at the SP10 and ST34 acupoints, following the principles of harmonized tonification and purgation. After obtaining *de-qi* sensation and curative effect, a 2 cm medicinal moxa stick (Nanyang Aiyang Moxa Products Co., Ltd., 3g × 54 pieces), with holes at the bottom, was lit and placed on the needle handle, maintaining a 2–3 cm distance from the skin to perform warm needling. The acupuncture was retained for 20 min. Patients should feel a comfortable warmth in the local acupoint during the treatment. If the patient experienced excessive heat, a cardboard shield was used to prevent burns. Needle operation was as above, not adding the moxa stick. Acupuncture treatment was performed for all subjects by the same licensed female acupuncturist of more than 10 years’ clinical experience. Except for acupuncture treatment, all participants did not receive any other treatment regimens. Acupuncture operators were only informed of the standardized operating procedures for each session without disclosure of specific grouping information. Assessors collected data under blind conditions, maintaining complete confidentiality of treatment assignment information throughout the entire evaluation process.

This study protocol was approved by the Medical Ethics Committee of The Third Affiliated Hospital of Fujian University of Traditional Chinese Medicine (Approval No. 2022-Kl-040). The study was conducted in strict accordance with the guidelines of the Declaration of Helsinki.​ The acupuncture interventions were conducted in strict compliance with the STRICTA (Standards for Reporting Interventions in Clinical Trials of Acupuncture) guidelines (MacPherson et al., 2010). It was registered (ChiCTR2400087846, 08/05/2024) before inclusion of the first participant.

Four different acupuncture schemes were tested over 4 separate days: (A) needle at SP10; (B) warm needle at SP10; (C) needle at ST34; (D) warm needle at ST34. Each protocol was spaced 5–7 days apart to accommodate participants’ schedules and minimize the carry-over effect of acupuncture therapy.

### 2.3 Data analysis

Normality of data distribution was assessed using the Shapiro–Wilk test, with ​homogeneity of variance verified by Levene’s test. Upon confirming that the data met both normality and homoscedasticity assumptions, a two-way analysis of variance (ANOVA) with repeated measures was performed to evaluate ​the hemodynamic effects of the two acupuncture methods (needle and warm needle) on the two specific acupoints (SP10 and ST34), including the main effects of the acupuncture method and acupoint selection, as well as their interaction effects. The significance level was set at P < 0.05. All statistical analyses were performed using SPSS 26 (IBM Corp., Armonk, ​NY, USA).

## 3 Results

### 3.1 Demographic information

The patients had the following characteristics (mean ± SD): age 62.79 ± 8.61 years, height 160.46 ± 0.51 cm, weight 57.25 ± 0.61 kg, BMI 22.22 ± 0.10 kg/m^2^, and disease duration of 35.08 ± 3.17 months (Table 2).

**TABLE 2:** Baseline characteristics of participants.

Female/male(n)	Patients with KOA (M±SD) 16/8
Age (years)	62.79 ± 8.61
Height (cm)	160.46 ± 0.51
Weight (kg)	57.25 ± 0.61
BMI(kg/m^2^)	22.22 ± 0.10
Course of disease(m)	35.08 ± 3.17

### 3.2 Hemodynamic alterations in knee osteoarthritis patients following acupuncture therapy: pre-post intervention analysis

**FIGURE 3:**
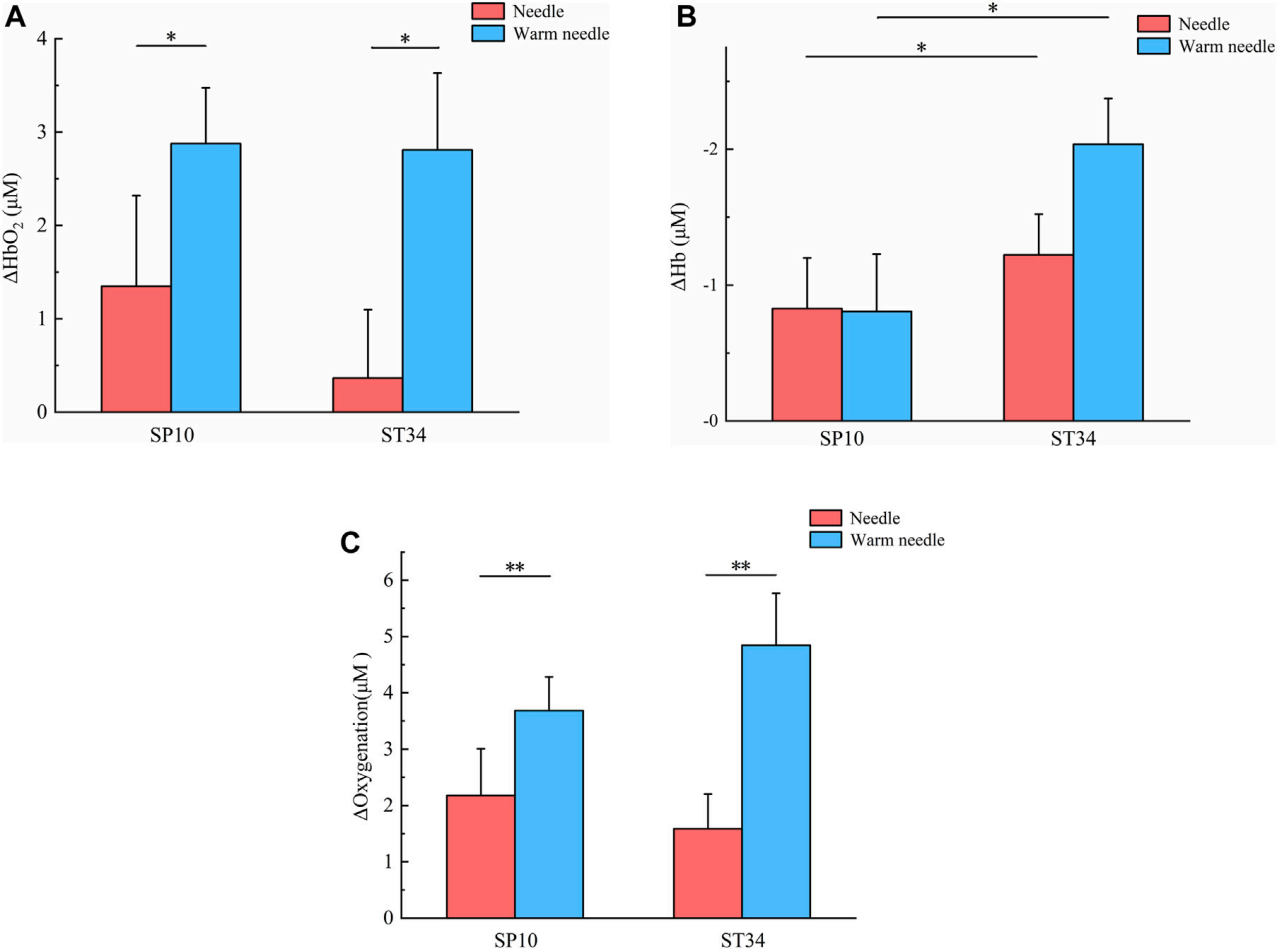
Changes in hemodynamic concentrations before and after four acupuncture therapy schemes. **(A)** Averaged oxyhemoglobin responses around acupoints, including skin and muscle, after four acupuncture therapy schemes. Values (mean ± SE) presented as the change compared to pre-acupuncture HbO_2_ value (Δ[HbO_2_]). * indicates significant difference of Δ[HbO_2_] between the two methods (P < 0.05). **(B)** Averaged deoxy-hemoglobin responses around acupoints, including skin and muscle, after four acupuncture therapy schemes. Values (mean ± SE) presented as the change compared to pre-acupuncture Hb value (Δ[Hb]). * indicates significant difference of Δ[Hb] between the two acupoints (P < 0.05). **(C)** Averaged oxygenation responses around acupoints, including skin and muscle, after four acupuncture therapy schemes. Values (mean ± SE) presented as the change compared to pre-acupuncture oxygenation value (ΔOxygenation). ** indicates significant difference of ΔOxygenation between the two methods (P < 0.01).

For HbO_2_, the results revealed ​no significant interaction between acupoints and methods (F = 0.334, p = 0.565). ​No main effect of acupoints was observed (F = 0.440, p = 0.509), whereas ​a significant main effect of methods was identified (F = 6.268, p = 0.014 and effect size = 0.064) (Table 3). For SP10, the change of oxyhemoglobin concentration in warm needle (2.87 ± 0.59 μM) was significantly higher than that in needle (1.34 ± 0.97 μM, P < 0.05). For ST34, the change in oxyhemoglobin concentration of warm needle (2.80 ± 0.82 μM) was significantly higher than that of needle (0.36 ± 0.73 μM, P < 0.05). Values are mean ± SE (Figure 3A).

**TABLE 3:** Statistical result of two-way ANOVA with repeated measures.

	*F* values	*P* values	Effect size
Acupoints			
HbO_2_	0.440	0.509	0.005
Hb	5.075	0.027*	0.052
tHb	1.890	0.173	0.020
Oxygenation	0.144	0.705	0.002
Methods			
HbO_2_	6.268	0.014*	0.064
Hb	1.209	0.274	0.013
tHb	2.659	0.106	0.028
Oxygenation	9.971	0.002**	0.098
Acupoints × Methods			
HbO_2_	0.334	0.565	0.004
Hb	1.345	0.249	0.014
tHb	0.002	0.968	0.000
Oxygenation	1.350	0.248	0.014

*P < 0.05,**P < 0.01.

For Hb, the results revealed ​no significant interaction between acupoints and methods (F = 1.345, p = 0.249). ​No main effect of methods was observed (F = 1.209, p = 0.274), whereas ​a significant main effect of acupoints was identified (F = 5.075, p = 0.027 and effect size = 0.052) (Table 3). For needle, the decrease of deoxyhemoglobin concentration in ST34 (-1.22 ± 0.30 μM) was significantly greater than that in SP10 (-0.82 ± 0.37 μM, P < 0.05). Similarly, for warm needle, the decrease in the deoxyhemoglobin concentration of ST34 (-2.03 ± 0.33 μM) was significantly greater than that of SP10 (-0.80 ± 0.42 μM, P < 0.05). Values are mean ± SE (Figure 3B).

For tHb, the results indicated no significant interaction between acupoints and method factors (F = 0.002, p = 0.968) and no main effects for either acupoint or method factors (F = 1.890, P = 0.173, and F = 2.659, P = 0.106, respectively) (Table 3).

For oxygenation, the results revealed ​no significant interaction between acupoints and methods (F = 1.350, p = 0.248). ​No main effect of acupoints was observed (F = 0.144, p = 0.705), whereas ​a significant main effect of methods was identified (F = 9.971, p = 0.002, and effect size = 0.098) (Table 3). For SP10, the change of oxygenation concentration in warm needle (3.68 ± 0.59 μM) was significantly higher than that in needle (2.17 ± 0.83 μM, P < 0.01). Similarly, for ST34, the change in oxygenation concentration of warm needle (4.84 ± 0.92 μM) was significantly higher than for needle (1.58 ± 0.61 μM, P < 0.01). Values are mean ± SE (Figure 3C).

## 4 Discussion

This study provides the first evidence that the effects of different acupuncture methods and acupoint selections exert distinct hemodynamic effects on local tissues in KOA patients. Compared to needle, warm needle significantly enhanced blood flow and oxygen delivery capacity (HbO_2_ and oxygenation). Both needle and warm needle interventions at ST34 demonstrated poorer oxygen consumption capability than SP10. Our results further revealed that blood flow and oxygen transport peaked 10 min after the acupuncture intervention. Warm needle maintained this peak state until needle withdrawal, whereas needle showed a continuous decline. Warm needle at SP10 induced a greater amplitude and longer duration of increased local oxygen metabolism than warm needle at ST34.​

**FIGURE 4:**
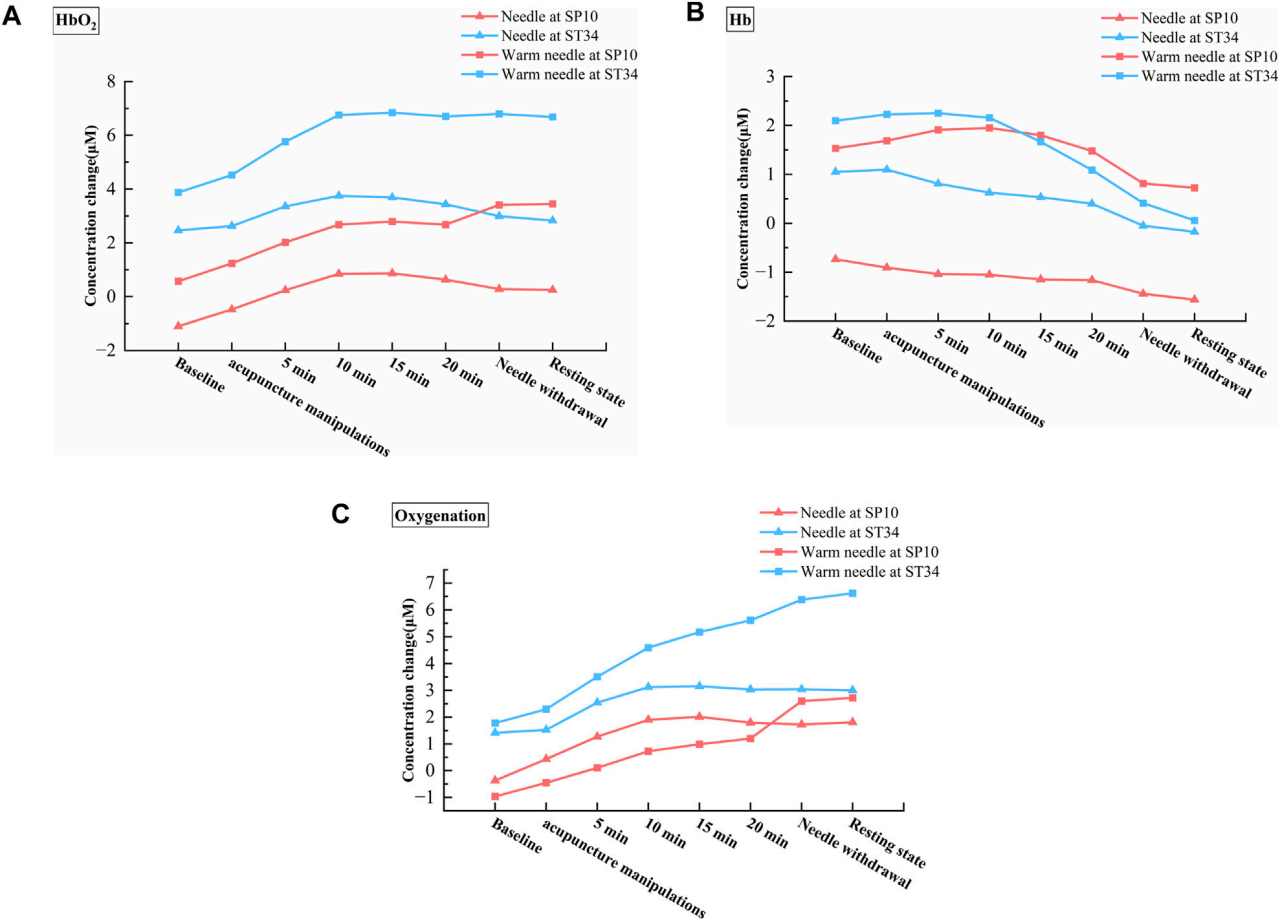
Hemodynamic changes across different phases of the four acupuncture therapy schemes. **(A)** Dynamic alterations in HbO_2_ concentration across treatment phases of the four acupuncture therapy schemes. **(B)** Dynamic alterations in Hb concentration across treatment phases of the four acupuncture therapy schemes. **(C)** Dynamic alterations in oxygenation concentration across treatment phases of the four acupuncture therapy schemes.

Using the non-invasive, optical device NIRS, the hemodynamic response to four different schemes of acupuncture therapy was quantified in this study for the first time. Our results indicated no significant interaction effects between acupuncture methods and acupoint factors on hemodynamic responses. However, significant main effects were observed: acupuncture methods exerted significant main effects on HbO_2_ and oxygenation, while acupoint selection showed a significant main effect on Hb. As illustrated in Figures 3A and C, under identical acupoint conditions, warm needle induced greater increases in HbO_2_ and oxygenation than needle. Figures 4A and C further demonstrate that for HbO_2_, both needle and warm needle interventions reached peak levels at 10 min post-intervention. Warm needle sustained this peak until needle removal, whereas the needle exhibited a gradual decline after 10 min. Notably, warm needle at SP10 continued to increase HbO_2_ even after needle removal, surpassing ST34. For oxygenation, needle intervention induced a marked increase at 10 min, followed by a declining trend after 15 min. Compared to baseline, warm needle achieved significantly greater oxygenation elevation than needle, with sustained increases persisting post needle removal.​ Oxygen is primarily transported in blood via binding to hemoglobin as oxyhemoglobin (Ciaccio et al., 2021); an elevated concentration of HbO_2_ indicates enhanced regional blood flow and oxygen delivery capacity. Oxygenation reflects the dynamic balance between oxygen delivery and consumption and cannot distinguish between the two (Li et al., 2023). These findings corroborate prior NIRS evidence showing significantly higher local HbO_2_ concentrations in KOA patients after FSN therapy with no significant increase in Hb, indicating enhanced blood flow and oxygenation (Yang et al., 2024).

These changes may be related to the mechanisms of acupuncture; however, the physiological mechanisms underlying its effects remain incompletely understood. Previous research has demonstrated that acupuncture treatment can modulate microcirculation in KOA patients (Min et al., 2015). Alterations in local tissue microcirculation are closely associated with vasoconstriction and vasodilation (Bagher and Segal, 2011; Gutterman et al., 2016). The participants in this study had long-term chronic knee osteoarthritis, and the research confirmed that acupuncture exerts anti-inflammatory effects. During this anti-inflammatory process, acupuncture induces the production and release of neurotransmitters and related peptides such as nitric oxide (NO), substance P (SP), and calcitonin gene-related peptide (CGRP) (Zijlstra et al., 2003). These substances, including NO, SP, and CGRP, can directly or indirectly act on vascular smooth muscle, inducing vasodilation and consequently increasing blood flow (Jou and Ma, 2009). Additionally, acupuncture modulates the autonomic nervous system by stimulating the vagus nerve to exert anti-inflammatory effects, reducing sympathetic nerve excitability, and promoting vasodilation (Lim et al., 2016). Vasodilation enhances blood perfusion and velocity, thereby improving oxygen delivery (Joyner and Casey, 2014). Warm needle, which involves attaching a burning moxa stick to the needle handle, has been shown to significantly increase local NO levels at acupoints compared to needle due to thermal stimulation, resulting in stronger effects (Hui et al., 2010). This leads to further peripheral vasodilation, increasing local blood flow and velocity. This study demonstrated that both needle and warm needle interventions induced maximal increases in local tissue blood flow and oxygen delivery 10 min after intervention. Tu et al. (2022) reported that 77.5% of patients experienced a 50% reduction in Visual Analog Scale (VAS) scores 10 min after acupuncture, with a 67.5% difference compared to sham acupuncture. This indicates that a 10-min acupuncture intervention significantly improves pain, consistent with our observed hemodynamic peak responses at this time point.​ Oxygenation levels depend on the difference between HbO_2_ and Hb concentrations and are positively correlated with changes in HbO_2_ concentration. This may explain why warm needle demonstrated higher concentrations of HbO_2_ and oxygenation than needle in the present study, as well as greater amplitude of concentration increases and earlier time to peak.​

Our study revealed an unexpected finding: both needle and warm needle interventions significantly reduced Hb concentrations at ST34 and SP10, with a greater reduction amplitude observed at ST34 compared to SP10 (Figure 3B). Furthermore, we observed that the concentration of Hb decreased continuously after needle intervention, while it decreased only after 10 min of warm needle. Notably, warm needle at SP10 induced a greater amplitude of Hb elevation and prolonged maintenance duration relative to warm needle at ST34 (Figure 4B). Hb originates from HbO_2_ after tissue oxygen consumption, reflecting local tissue oxygen metabolism capacity (Gao et al., 2023).

These findings may be associated with chronic inflammatory-induced soft tissue atrophy, unique hemodynamic alterations in KOA patients, and acupoint-specific properties. Studies indicate that KOA patients exhibit quadriceps muscle atrophy with reduced muscle fiber cross-sectional area (Fink et al., 2007; Mau-Moeller et al., 2017), leading to decreased mitochondrial density, impaired ATP synthesis efficiency, and diminished oxygen consumption (Conley et al., 2000; Di Felice et al., 2022). Additionally, KOA-related arterial abnormalities correlate with disease progression, characterized by accelerated lower limb arterial flow velocity but reduced effective perfusion—the “high-flow, low-perfusion” phenomenon—which exacerbates muscle damage and metabolic dysfunction (Fukumoto et al., 2021; Liu and Li, 2017; Wu et al., 2023). We hypothesize that while needle increases blood flow and oxygen delivery at SP10 and ST34, its capacity to enhance local tissue metabolic activity remains limited. In contrast, warm needle not only improves oxygen delivery but also transiently elevates local oxygen consumption and metabolic function, albeit with short-term sustainability. The 2021 Nobel Prize in Physiology or Medicine findings further elucidated the therapeutic mechanisms of acupuncture: needle stimulation activates mechanoreceptors, while warm needle concurrently activates both mechanoreceptors and thermoreceptors (Guo et al., 2022). The biphasic trend of Hb concentrations in warm needle (peaking at 5 min post-intervention for ST34 and 10 min for SP10) may be related to temperature dynamics during warm needle. ​Moxa combustion elevates needle temperature up to 48 °C, with thermal stimulation exceeding 43 °C activating ​​transient receptor potential vanilloid 1 (TRPV1)​ receptors (Bang et al., 2023; Caterina et al., 1997). TRPV1 activation triggers calcium/sodium influx, membrane depolarization, and ATP synthesis/release (Chen et al., 2018). During warm needle intervention, the needle temperature initially rises continuously and gradually decreases as the moxa cone burns. This may explain why warm needle enhances local tissue oxygen consumption capacity while exhibiting a limited maintenance duration.​

According to classical Chinese medicine (CM) theory, KOA falls under the category of “*bi* syndrome”, characterized by a deficiency-rooted excess pathogenicity and intertwined deficiency-excess patterns. Acupuncture regulates *qi* and blood circulation while balancing *yin* and *yang* (Wen et al., 2021). Warm needle therapy integrates the mechanical stimulation of acupuncture, the thermal effects of moxibustion, and needle-mediated heat conduction. Heat stimulation likely activates thermoreceptors, thereby dispelling wind-cold pathogens and harmonizing *yin–yang* (Zhang and Gao, 2018), which may explain the superior hemodynamic changes in warm needle at SP10 compared to needle intervention. Additionally, KOA patients exhibit atrophy and weakness of the vastus medialis (VM) and stiffness of the vastus lateralis (VL) (Chang et al., 2021; Shen et al., 2024). SP10 is located at the VM along the Spleen Meridian of Foot-Taiyin, while ST34 resides in the VL along the Stomach Meridian of Foot-Yangming (Chang et al., 2021). According to CM theory, “The spleen governs the muscles and limbs”. Spleen deficiency leads to muscular atrophy and weakness. For *yin* meridians, warm needle enhances *qi* and blood replenishment due to their thermal properties. Therefore, warm needle at SP10 induces more favorable oxygen metabolism alterations than warm needle at ST34.

The application of NIRS in acupuncture for KOA has been increasingly studied; however, current research predominantly focuses on cerebral hemodynamic changes, while peripheral local tissue hemodynamics remain underexplored. As a preliminary exploratory study, this trial has several limitations. First, its design primarily investigated local tissue hemodynamic responses pre- and post-intervention across four treatment protocols and dynamic changes during acupuncture phases but failed to clarify the long-term effects of these interventions on improving local tissue hemodynamics.​ Subsequent studies should implement randomized controlled trials with multiple timepoint monitoring to systematically evaluate temporal efficacy profiles. Second, the gender ratio imbalance in participants leaves it unclear whether gender factors influence experimental outcomes. Future recruitment should optimize gender distribution to control sex-related confounders. Finally, potential bias arising from heterogeneity in disease severity among participants necessitates stratified subgroup analyses based on Kellgren–Lawrence grading to enhance the reliability of studies.

## 5 Conclusion

This study provides the first evidence showing the effect of acupuncture methods and acupoint selections of acupuncture therapy on hemodynamic responses in 24 KOA patients. The results indicate that warm needle significantly improves local blood flow and oxygen delivery capacity in KOA patients, extending the duration of improvement effects more effectively than needle intervention. Warm needle at SP10 demonstrated superior efficacy in improving oxygen metabolism relative to warm needle at ST34.​

## Data Availability

The raw data supporting the conclusions of this article will be made available by the authors, without undue reservation.
